# Nurses experience increased clinical and organisational competence by working with a medical quality register, RevNatus – a qualitative study

**DOI:** 10.1186/s12913-022-08595-x

**Published:** 2022-10-26

**Authors:** Hilde Bjørngaard, Hege Svean Koksvik, Bente Jakobsen, Kjersti Grønning

**Affiliations:** 1 Norwegian National Advisory Unit on Pregnancy and Rheumatic Diseases, Department of Rheumatology, Trondheim University Hospital, St.Olavs Hospital, 7030 Trondheim, Norway; 2 Department of Public Health and Nursing, Faculty of Medicine and Health Sciences, Norwegian University of Science and Technology, Trondheim, Norway; 3 Nord-Trøndelag Hospital Trust, 7601 Levanger, Norway

**Keywords:** Nursing, Rheumatology, Pregnancy registry, Qualitative research, Outcome measures

## Abstract

**Background:**

RevNatus is a consent-based, nationwide medical quality register that collects data on patients with inflammatory rheumatic diseases during pregnancy and one year postpartum. The entering of data takes place in outpatient clinics in rheumatology wards in hospitals. The aim of this study is to explore how rheumatology nurses experience organizing and working with the medical quality register RevNatus in addition to their normal clinical patient-care tasks.

**Methods:**

Qualitative focus group interviews and individual in-depth interviews were conducted in 2018 to gain insights into how nurses organize performing quality register work and clinical work simultaneously. Data were analysed using systematic text condensation.

**Results:**

The informants represented seven different rheumatology outpatient clinics in Norway. The analyses showed that working with RevNatus increased the nurses’ knowledge about pregnancy and rheumatic diseases, improved the content of their nurse consultations and found the ‘register form’ as a useful template to structure the nurse consultations. The nurses took the main responsibility for RevNatus, but lack of routines and uncoordinated collaboration with the rheumatologists and secretaries made the nurses spend too much time verifying the accuracy of data or post-registering missing data.

**Conclusion:**

The nurses experienced work with RevNatus as time-consuming, but the register work increased both their clinical and organisational competences. Routines and collaboration within the registry team are important to ensure the data quality and reduce the workload.

**Supplementary Information:**

The online version contains supplementary material available at 10.1186/s12913-022-08595-x.

## Introduction

There are more than 200 different rheumatic diagnoses, many of which affect women of fertile age. Most rheumatic diseases are chronic, and several cause stiffness and pain in joints and muscles, but some also affect the skin, lungs, mucous membranes and other organs [1]. Depending of severity of disease and disease activity before pregnancy, some women with rheumatic disease have increased disease activity during pregnancy, while others experience increased disease activity postpartum [2–7]. The disease itself and the medical treatment can affect pregnancy and adverse interactions may occur [8, 9]. Pregnancies in women with rheumatic disease are differentiated as high or low risk depending on the diagnosis [10]. Diseases with a low risk for complications during pregnancy are diagnoses like Rheumatoid arthritis, Spondyloarthritis, Psoriatic arthritis and Juvenile rheumatoid arthritis [3–5, 7]. Diseases that affect connective tissue or blood vessels like vasculitis put women at high risk for complications during pregnancy [6, 8]. The risk has an impact on the frequency of the follow-ups throughout pregnancy [10]. National guidelines recommend regularly scheduled appointments at rheumatology outpatient clinics throughout pregnancy and the first year after childbirth to monitor disease activity or flare-ups and rheumatic medications [10, 11]. The medical quality register RevNatus was established to monitor treatment, disease activity and patient follow-up during pregnancy and the postpartum period. The purpose of RevNatus is to obtain knowledge about the interaction between pregnancy and rheumatic disorders, treatment during pregnancy, disease activity and pregnancy outcomes [12, 13], and to ensure high quality treatment and follow-up of patients with inflammatory rheumatic diseases who plan pregnancy or who are pregnant [13].

There are over 50 national medical quality registries in Norway [14]. The medical quality registries in general aim to facilitate quality improvement [14, 15], elucidate professional roles and improve clinical care [15]. Furthermore, medical quality registers are a structured collection of medical information about the assessment, treatment and follow-up of patients [14]. They can be linked to specific incidents, like an operation or intervention, while others monitor patients’ pathways and collect data during the patients’ follow-ups [16]. The purpose of collecting data in quality registers is to obtain knowledge about a specific group of patients, monitor the quality of treatment, and report results to initiate quality improvement [14]. The quality of the data is vital for a medical quality register [14, 17]. Insufficient data quality is often associated with healthcare workers’ lack of motivation in collecting and validating the accuracy of data, lack of training, uncertainty regarding definitions of variables, or incorrect typing [18]. Training those who register data is crucial to the data quality [19, 20]. Several challenges related to prioritization between work with patients and work with the register may occur when healthcare workers need to execute register tasks and patient care simultaneously without extra resources for the tasks [21]. Healthcare workers state that registry work is time-consuming, and they are concerned that the increased workload without increased resources or redesigned practice may affect patient care [21, 22].

It is, however, recommended that register work is implemented as part of the daily routines in the clinic [21, 23]. Nurses are often responsible for entering data in the registries [15, 21], but the role of a nurse is complex and the tasks are not always clearly defined [24]. There has been a shift in nurses’ tasks from patient-oriented bedside duties to more organizational tasks where nurses have to manage both good patient care and organizational efficiency and productivity [25], often referred to as ‘the invisible work of nurses’ [24]. According to the recommendations from the European Alliance of Associations for Rheumatology (EULAR), nurses have been assigned increased responsibilities in the follow-up of patients with rheumatic diseases [26]. They have undertaken new tasks that require updated clinical knowledge and skills [25, 26], such as registry work, and their extended roles have had a positive influence on patients’ satisfaction with care [27].

Due to nurses ‘extended roles requiring both updated clinical knowledge and skills [26, 28] and organizational competence, more knowledge is needed on how nurses experience combining clinical and organizational work [24, 25, 29]. Previous studies have not explored how nurses experience working with a quality register simultaneously as doing clinical work and caring for other patients. Hence, the aim of this study is to explore how rheumatology nurses experience organising and working with the medical quality register RevNatus in addition to clinical patient-care tasks.

## Methods

A qualitative approach using semi-structured focus group and individual in-depth interviews was chosen to gain in-depth insights into how rheumatology nurses experienced organising and working with RevNatus in addition to clinical patient-care tasks. Qualitative methods are well suited for exploring common experiences from a particular area or phenomenon [30]. Group interviews are suitable for facilitating interaction amongst the nurses, while individual interviews assist with gaining an understanding about each nurse’s own experience.

An e-mail was sent to a strategic sample of 24 nurses in Norway in 2018, inviting them to participate in the focus group interviews. Eight nurses provided a written informed consent to participate. One focus group consisted of five nurses and the other had three nurses, altogether representing seven different hospitals. The interviews lasted between 46 and 49min and were conducted at a conference hotel. Additionally, four nurses consented to participate in individual telephone interviews that lasted between 23 and 29min. All interviews were conducted from April to May 2018. A trained interviewer familiar with RevNatus was a moderator in the focus group interviews, while the first author took notes. The individual interviews were conducted by the first author. All interviews were audiotaped and transcribed verbatim. The interview guide was developed by the first author in collaboration with all the authors and was based on the research question and a literature review about quality registries and nurses’ roles. The focus group interview guide contained questions about how the nurses experienced working with the quality register, their routines, their training and how they organized the registry work alongside other tasks to be done in the clinic. The focus group interviews were used as a background to sharpen the interview guide for the individual interviews [31]. Before conducting the individual interviews, the authors reviewed the focus group interviews to improve the individual interview guide, and the first author adjusted the original interview guide.

### Setting

Data for RevNatus are provided by 19 rheumatology units in Norway. The inclusion criteria are an inflammatory rheumatic disease, a pregnancy-wish and a written consent to be included in the register. The register has a high grade of national coverage, as it includes register data from 19 of the 22 rheumatology units in the country. The register is now web-based, though previous versions were paper-based. The data collection, which is a part of the rheumatology outpatient consultations, is carried out by a nurse or the rheumatologist, in accordance with national guidelines [10]. The purpose of the rheumatology outpatient consultations during pregnancy and postpartum is to observe and control the disease activity, adjust medical treatment, and detect and treat any complications that may occur due to rheumatic disease [2, 8, 10, 11, 32].

### Ethics

The study was assessed by the Norwegian Centre for Research Data (NSD – nr 58,634) and approved by the Research Committee at St Olavs University Hospital, Norway. Information that can identify departments or persons and quotes have been anonymised.

### Analyses

The data were analysed using systematic text condensation (STC), a modification of Giorgi’s phenomenological method. Essential steps in STC were to get a sense of the whole material, to identify and sort meaning units, to transform and abstract meaning units and to synthesize the meaning units into consistent statements [33]. The first author identified, coded and sorted the meaning units into categories and linked the categories to the preliminary themes. The authors discussed and refined the categories during several meetings. Then, the first author condensed and summarized the data into generalized descriptions of how the nurses´ experienced organizing and working with the medical quality register RevNatus in addition to clinical patient-care tasks, described as the final categories. The analyses were conducted as collaborative negotiations between the authors. All the authors read all the interviews to achieve a nuanced perspective on the analysis and reduce the risk of single-researcher preconceptions. The first author validated the interpretations and findings against the initial transcripts to ensure that the synthesized result reflected the original context. An example of the analysis process is illustrated in Table 1.

**Table 1:** Example of the step-by-step process of analysis using systematic text condensation

Step 1	Step 2	Step 3	Step 4
**Preliminary category**	**Meaning units**	**Condensation**	**Final category**
Meaningful knowledge	“Because of RevNatus we obtained knowledge of the group of patients and of the treatment, which increased the knowledge in the unit as a whole”… “In a way, knowledge comes from experience from following these patients, from having the overview of when things turn out well or not, in addition to research.”	The registry contributed to increased knowledge, which subsequently provided support during patient conversations.	Increased competence

## Results

The nurses represented seven different rheumatology outpatient clinics in Norway, both small and big units, and all informants had a formal local role related to RevNatus, see Table 2.

**Table 2:** Informants

Interview	Region	Big Unit > 150 registrations in 2018	Medium unit > 75 registrations in 2018	Small unit < 75 registrations in 2018
Focus group 1	Health in South East part of Norway	2 nurses		1 nurse
	Health in Western part of Norway			1 nurse
	Health in Middle part of Norway		1 nurse	
Focus group 2	Health in Western part of Norway	1 nurse		
	Health in South East part of Norway		1 nurse	
	Health in North part of Norway		1 nurse	
Individual in-depth interview	Health in South East part of Norway	1 nurse		
	Health in Western part of Norway		2 nurses	
	Health in Western part of Norway	1 nurse		

An overview of the results is illustrated in Table 3. The main result was that the nurses experienced an increase in knowledge about pregnancy and rheumatic diseases as a result of working with the quality register RevNatus. This competence strengthened the quality of the nurses’ consultations with the women, while they also discovered that the ‘register form’ was a useful template to better structure the content in the consultations. The analyses further showed that the nurses took the main responsibility for making sure that necessary data were registered in RevNatus. Thus, more collaboration and better teamwork between the nurses and rheumatologists would improve the organising of the registry work, making it less time-consuming for the nurses who spent time correcting, validating and post-registering missing data. The results are further presented as four main categories with their subcategories below.

**Table 3:** Categories

Research question	Final categories	Subcategories
How do nurses experience working with the quality register RevNatus in addition to clinical patient-care tasks	Increased competence	Increased knowledge about pregnancy and rheumatic diseases Improved consultations Initial training is necessary
	Routines and priorities	Challenging logistics with appointment scheduling Dilemmas in prioritizing between patients Lack of collaboration and teamwork
	Responsibility	Establish a good patient flow Ensure high data quality Remind the doctors

### Increased competence

The informants reflected on several positive effects of working with RevNatus. The registry work resulted in an increased focus on pregnancy and pregnancy-related issues in women with rheumatic diseases in the clinics, and the healthcare providers learned more about pregnancy, rheumatic disease and disease activity measures. The registry work emphasized patients` needs for follow-up during pregnancy as according to national guidelines. The informants said that they gained in-depth knowledge about the interaction between pregnancy and rheumatic diseases, the disease course during and after pregnancy, and pregnancy outcomes. In addition, the informants reported that the registration form was an excellent template for structuring the consultations with the women. The questions in the registration acted as a checklist for important aspects necessary to monitor during pregnancy, such as abnormal blood tests, pain, fatigue and symptoms of increased disease activity.

I feel that we are contributing to an increase in the clinic’s competence and are raising the awareness of this group of patients with tighter follow-up and that we are getting better training. (Nurse, focus group 2)

The informants further said that their impressions were that the women were satisfied with the follow-up regime. The tight controls made them feel safe, and they were happy to provide data for RevNatus, even if they had to answer several questions about their pregnancy and health status at every visit.

Most of our patients are unsure about what will happen during the pregnancy, so they feel safe with the follow-up. (Nurse, individual interview 2)

Some of the informants said they became very well acquainted with their patients by monitoring the women’s disease activity and health status throughout the pregnancy and first year postpartum. Some of the nurses also gave the women ‘general pregnancy advice’ – e.g., how to ease their pain if they couldn’t use analgesics during pregnancy – in addition to collecting data for RevNatus.

This is the closest I will ever be to my dream of being a midwife! (Nurse, group interview 1)

When the informants were asked about training before they started to work with and register data in RevNatus, they mentioned different kinds of initial training. Some said that their ‘basic’ knowledge about registers was insufficient when they started to register data in RevNatus, but their knowledge improved quickly by ‘learning and doing’. Others felt particularly uncertain about how to correctly enter the variables in RevNatus – e.g., the blood and urine samples –while others were unsure about how to measure ‘disease activity’, how to set a value on ‘damage indexes’ or how to deal with different diagnostic criteria.

It should have been clear in the medical records whether the patients are filling Caspar or other criteria, but I have to try to figure it out for myself. I can’t guarantee that it’s always right. I’m doing my best. (Nurse focus group 1)

Everyone believed, however, that it was easy to get in touch with the office staff for RevNatus for guidance and support with potential questions.

Sometimes I don’t bother looking it up, I call or send an email instead, and get a response straight away. Their availability is very good. (Nurse focus group 1)

### Routines and priorities

Many of the informants expressed the feeling that keeping track of the follow-up appointments and obtaining patients’ written consent to participate in RevNatus was challenging, due to the lack of routines governing ‘who is responsible for what’. Since RevNatus is a comprehensive and complex registry that requires knowledge about what data to collect at what time (before, during or after pregnancy?), some informants experienced a shift in personnel as another challenge. The informants thought that the personnel at the clinics were inadequately familiar with RevNatus due to how the clinics organised the RevNatus work. At some clinics, the nurses found it difficult to coordinate appointments according to the recommended follow-ups, often resulting in additional work to control scheduled appointments. To keep track of the logistics, the nurses created their own systems to secure the flow of patients.

It’s perhaps the most challenging thing about the job, I think, that I actually have to control that the patients get appointments at the right time even if it is scheduled by the recommendations [and she continues], the logistics of the patients’ appointments are perhaps the most challenging thing, more than the actual registration once they’re here. (Nurse individual interview 1)

A mutual challenge experienced by all the informants was lacking routines for how to enrol patients in RevNatus before they become pregnant and getting complete registrations throughout the entire register period. The informants mentioned poor communication between doctors and nurses, insufficient knowledge of RevNatus’ existence among all the healthcare personnel, and not having RevNatus registry work implemented in daily routines as possible reasons for missing enrolment prior to an established pregnancy.

Sometimes, it could take some time. Then when they come to a doctor who knows RevNatus, who asks why they are not in RevNatus, then they include them, but then it is a little late. (Nurse individual interview 1)

The informants said that working with RevNatus by collecting data at the right time, entering the accurate data, collecting patients’ reported data and having the right blood tests taken, was time-consuming. Several of the nurses had established their own control routines to keep track of when to register data in RevNatus, and if a patient did not meet as scheduled, the nurses strived to obtain the necessary information needed for the register.

If they don’t live that far away, we ask them to stop by and take the blood tests and talk if they are in town. (Nurse in focus group 1)

When talking about clinical work and collecting data for RevNatus, the informants sometimes saw it as a dilemma that ‘women in remission’ (i.e., with low disease activity) were prioritized in the clinic at the expense of other non-pregnant patients with high disease activity. Some of the clinics prioritized RevNatus and consultations for all the women included in RevNatus independent of ‘remission or not’, while others found it challenging to prioritize consultations for women in remission because of a shortage of available appointments. The head of one of the clinics had decided not to schedule consultations if the patients were in remission. One of the informants also said she thought it was a waste of resources to schedule appointments if the only purpose was to collect data for RevNatus.

If you give the patients an appointment only to register in RevNatus, should you take that assessment and tell the coordinator and constantly push for them to be prioritized? It can be difficult when you know that the resources are limited. (Nurse individual interview 1)

The informants agreed that collecting and entering data in RevNatus was time-consuming in addition to other clinical tasks. Even though the registrations should take place at the same time as the outpatient consultation, several informants experienced that this was not the case. Some clinics prioritised register work because the required extra resources existed, while others did not. On busy clinical days, several of the informants said it was challenging to find enough time to complete the RevNatus forms, for example, to search for correct information in the patient record system and find historical data about medication or diagnosis criteria.

If you have to look in old records for information about disease onset or on what criteria the doctor based the diagnosis, you can spend a whole day searching. (Nurse focus group interview 2)

### Taking responsibility

Some of the informants said that they felt an obligation to take the main responsibility for RevNatus because communication within the team about ‘who was responsible for what’ was lacking. Sometimes, the informants had to complete missing registrations, and they found the register’s mandatory variables valuable since they acted as reminders for missing data before closing the register. Often the nurses had to remind the doctors about RevNatus because lacked awareness of the registry and women with a planned pregnancy to consent to the registry.

I feel that the doctors don’t have RevNatus in mind. We have to write notes to remind them before appointments with patients. (Nurse individual interview 2)

The informants said that they prioritised taking time to contribute ‘high quality data’ in RevNatus, even if it was time-consuming and occasionally at the expense of other tasks. The informants therefore spent a lot of time checking for or completing missing data, such as whether lab results were available or not prior to the consultation. Completing data in retrospect could be a source of error if it was done in a rushed manner.

It takes a lot of time to register in RevNatus and when you consider that we spend a lot of time doing that, it is important that the work we do is correct or else it is somehow useless. (Nurse individual interview 4)

The informants also explained that they took responsibility for educating new nurses when there was a shift in key personnel. They felt obligated to train new nurses because sufficient knowledge is necessary for knowing which data to collect, how to make the registrations correct and how to prevent missing data.

We talk together, support and help each other if we encounter something difficult or problematic. (Nurse individual interview 4)

## Discussion

This study shows that the nurses experienced an increased knowledge about pregnancy and rheumatic diseases due to the register work in RevNatus. Their increased knowledge strengthened the quality of their consultations with the women, and they experienced the ‘register form’ as a useful template to better structure the content in their consultations. The results further showed that the nurses took the main responsibility for registering data in RevNatus. However, the RevNatus registry work could have been better organised with more collaboration and teamwork between the nurses and rheumatologists. Better teamwork could have influenced the registry work in a positive manner and made the registry work less time-consuming.

### RevNatus in the clinic

Research shows that healthcare personnel are sceptical about implementing quality registry work alongside clinical work because they fear an increased workload without increased resources [22]. Our findings confirm that it is time-consuming to facilitate data collection in addition to other patient follow-up tasks, especially if the register work is not implemented in the organization and not sufficiently known by the professionals [21, 34]. Another study aiming to explore assistant nurses’ perceived impact on their work situation found that performing assessments and registrations in registries added a time-consuming task to their regular work routines, which also required certain computer skills. But the registry work also led to a perception of a decreased workload and reduced stress because the registry work contributed to clarification of work roles [15].

This study further shows that the nurses were dedicated, as they demonstrated by taking the main responsibility for making RevNatus a register with high-quality data. They pushed the registry forward and felt that the doctors took a passive role. However, to achieve a registry with high-quality data, the professionals working with the register need to collaborate [34, 35]. They need to decide each team member’s role and responsibility for collecting and entering data. Although other studies have shown that registry work could lead to increased collaboration across professions, [15, 35] the current study did not.

The logistics of recording data from patients at predefined times was challenging. For example, when patients had an appointment rescheduled without notifying the nurses, the registrations in RevNatus were missing. When this occurred, the nurses often had to enter data in retrospect based on the patient’s record, confirming the findings from other studies that the workload increases if the register work is not integrated into the daily routines [15, 21, 36, 37]. If the web-based forms have not completely replaced paper forms, the data-entry workload will be dual [21, 36, 37]. When the nurses in this study used paper forms instead of entering data directly into the web-based forms, they did it because the awareness of RevNatus was insufficient among doctors in the department. The nurses placed the paper on the doctor’s desk, collected the paper after the consultation and then entered the data on the web. Entering data from a paper registration into a web-based system increases the risk of accidental errors [18].

The results further showed that the nurses created their own local systems to ensure patient follow-up and proper patient flow because formal routines, collaboration and teamwork were.

lacking. Lack of administrative planning when implementing registry work in addition to other clinical tasks will by default make registry work an individual responsibility [21].

However, working with the quality register contributed to increased quality of the nurses’ consultations [15] as the nurses gained more knowledge about pregnancy and rheumatic diseases, and they used the ‘RevNatus form’ as a template in the consultations. If the registry contains updated and available data during the consultation, the register can also be actively used during the consultations to inform the patient about her ‘pregnancy trajectory’ [38], involving the patient more closely in the treatment and follow-up [39]. Other studies have also shown that working with a quality register has several positive implications [15, 35]. The health care personnel in one study explained that the registry work increased their willingness to learn new things by taking on more responsibilities and increased reflection due to the clear and available documentation [15]. A different study discovered that the health care personnel created new ways for the talking with patients due to the registry work implications [35].

This study further showed that the hospitals organized the registry work, clinical follow-up and the data collection differently. Several nurses explained that they did not have any training or education when they started to work with RevNatus. This study was not able to fully detect why the hospitals organized the work differently, because we did not interview the managers. However, lack of planning, managerial support and clarification of roles are shown to be factors influencing the implementation of quality registries in the clinic [21]. Even though the hospitals organised the RevNatus registry work differently, the nurses took overall responsibility for the registry, confirming findings from another study of registry work in cardiac rehabilitation where the nurses showed a similar sense of shared responsibility [21].

### Nurses’ dual competence

The informants in this study were ‘contact nurses’ for RevNatus, but the contact nurse role was not clearly defined. The nurses discussed how complicated it could be to provide care to patients with a complex disease, to monitor the disease and the pregnancy, and to manage the registry work as well. This was especially the case when dealing with the invisible organisational tasks [29], such as controlling scheduled consultations, securing patient flow and checking patient records, which altogether illustrates the “nurses’ dual competence” [24, 25]. The dual competency – ‘administrative /organisational’ and clinical competence – is needed to create fluency in the work processes [24, 25]. The nurses in this study used both their professional clinical competence and organizational competence when scheduling appointments, deciding whether the data collection should be done by telephone or a physical visit and performing clinical assessments of the women’s conditions [25, 26].

The findings also showed that some informants did not have sufficient knowledge in rheumatic diseases, medical treatment, pregnancy, registry work [18, 19] and organizing health-care follow-up [25] when they started to register data in RevNatus, but they learned quite a lot when they began to work with the registry. As it was obvious that a lack of knowledge may lead to errors when entering data in a registry and affect the validity of data [40], initial and follow-up training was very important to ensure that the data quality in a register stays high [19, 20].

### Strengths and limitations

This is the first study exploring how rheumatology nurses experience the organizational work with a medical quality register in addition to providing clinical care to patients in a Norwegian setting. To achieve trustworthiness (credibility, dependability and transferability) as described by Granheim and Lundeman [41] the chosen context was rheumatology departments. We recruited nurses with different RevNatus work-experiences from several hospitals across Norway as informants. The informants had both experiences from RevNatus registry work and caring for pregnant women in the outpatient clinic. To make sure that the informants could speak freely about their experiences, a nurse with many years of clinical experience in caring for pregnant women with rheumatic diseases and trained in conducting qualitative interviews performed the focus-group interviews. The focus group interviews were used as a background to sharpen the interview guide for the individual interviews. To prevent that the individual researcher preconception should affect the results, all the authors joined the analysing process. To ensure the dependability in the study and prevent the risk of inconsistency during data collection, all interviews were conducted during a period of three months. Another strength of this study is the transferability of the findings. Even though the study has explored nurses’ experiences within a narrow medical field – pregnancy and rheumatic diseases – the findings are transferable to other medical settings combining clinical patient care with registry work. Good planning and managerial support is important if nurses should include registry work in addition to patient-care tasks as one of several tasks. Ensuring that registry tasks and responsibility are shared among the clinicians, that registry work becomes part of the daily routines in the department, and that the personnel are sufficiently trained are also findings transferable to other clinical settings and registries for patients with other diagnoses.

A noteworthy limitation in this study is that the interviewer for the individual interviews had a formal role in the administration of RevNatus, making it possible that the informants held back information that was not beneficial for RevNatus or they did not speak freely about how they experienced working with RevNatus.

## Conclusion

Working with the medical quality registry RevNatus increased the nurses’ knowledge about pregnancy and rheumatic diseases. The increased knowledge improved the nurse consultations, and the ‘register form’ was a useful template for structuring the consultations.

The contact nurses took the main responsibility for the register work to ensure the data quality of the register. When implementing registry work in addition to patient-care tasks, it is important that the implementation is planned, that the registry tasks and responsibility are shared, that registry work becomes part of the daily routines in the department and that the personnel are sufficiently trained.

## Electronic supplementary material

Below is the link to the electronic supplementary material.

Supplementary Material 1

## Data Availability

The data supporting this study?s findings are available from the corresponding author upon reasonable request.
