# Supplementary material for: Nurses experience increased clinical and organisational competence by working with a medical quality register, RevNatus – a qualitative study
Source: BMC Health Serv Res. 2022 Oct 26;22:1291. doi: 10.1186/s12913-022-08595-x (PMC9608925; doi:10.1186/s12913-022-08595-x)
Supplement: Supplementary file 1 — Supplementary Material 1 [file 12913_2022_8595_MOESM1_ESM.docx]

Appendix 1

Interview guide

| Group interviews | Individual interviews |
| --- | --- |
| How do you find the process of recording data in RevNatus? | What experiences have you made working with RevNatus, both as pertain to general knowledge and in training situations? |
|  |  |
| Can you briefly tell us how you coordinate the work with RevNatus in relation to other tasks, procedures, routines etc. ? | Can you say something about the organization of work tasks in your workplace, in relation both to communication and collaboration? |
| Can you elaborate on the training, if any, you received prior to starting? | Do you have any thoughts on the data quality in RevNatus? Completeness or missing data? |
| Do you experience having sufficient knowledge and expertise to carry out registrations, or have you noticed deficiencies in certain areas? | Can you say something about the usefulness of RevNatus? |
| Do you find the work of data entry in RevNatus to be useful ? | Other things? |
